# Why the COVID-19 Crisis Is an Ethical Issue for Business: Evidence from the Australian JobKeeper Initiative

**DOI:** 10.1007/s10551-023-05392-2

**Published:** 2023-03-24

**Authors:** Graciela Corral de Zubielqui, Howard Harris

**Affiliations:** 1 Adelaide Business School, The University of Adelaide, Adelaide, SA 5005 Australia; 2 UniSA Business, University of South Australia, Adelaide, SA 5000 Australia

**Keywords:** Ethical decision-making, Organisations, JobKeeper, Public pressure, Crisis

## Abstract

The COVID-19 virus was unveiled to the world as a health crisis and later also as an economic crisis. For some organisations, it has become an ethical crisis. This is certainly the case for large organisations in Australia, where the way many enterprises handled a government wage subsidy called JobKeeper led to a public backlash, media pressure, and a variety of responses ranging from ‘We acted legally’ to the full return of the subsidy. Some organisations later reported profits, and the public response indicated concern about this behaviour, many considering it immoral despite it being legally compliant. It is, we contend, a question to which stakeholder theory can be applied, examining how organisations view and respond to the public. We use content analysis of mainstream media to provide information about public reactions and information from official sources to confirm corporate action. We show that there is a significant ethical component in the public response to the behaviour of organisations as they respond to the crisis. COVID has been an ethical, health, and financial crisis for these organisations. Public pressure, exerted in and through the media, made the general public a definite stakeholder.

## Introduction

The COVID-19 pandemic has been seen as a crisis. First, it was considered a health crisis; then, governments and others quickly labelled it an economic crisis (Douglas et al., 2020). However, for some organisations, it has become a business ethics crisis. This is especially true with regard to the behaviour of some large organisations that received government assistance due to the pandemic. This study describes a support program in Australia (JobKeeper) that was intended to subsidise employee wages and salaries during the downturn and shows how COVID-19 came to be a perceived ethical issue for businesses.

It became apparent that some support recipients reported profits, which prompted some to perceive that these firms had acted unethically. This paper focuses on the perception or appearance of ethical wrongdoing rather than a normative analysis. We identify policy responses to the COVID-19 crisis related to business ethics and public responses to companies receiving money from the JobKeeper program, particularly in the aftermath when the public began to view the fund provision as unnecessary. We have examined the public response and determined which ethical concerns were most frequently mentioned, drawing attention to the fact that something is valid within the legal context might still be perceived as ethically wrong by a different stakeholder.

We build our case by reviewing media coverage over two years, using as a framework business and management literature that has explored business decision-making and ethical issues (Jones, 1991; Treviño et al., 2006; Zeni et al., 2016), stakeholder theory (Freeman, 1984) and business decision-making models (Jones, 1991; Rest, 1986). Despite extensive review studies on stakeholder theory and business decision-making, and ethical issues within the business and management discipline, there is a lack of understanding of how ethical decisions play a role under crisis circumstances, and there is little about the role that the public can play as stakeholders in influencing decision-making (apart from the study reported in Dorobantu et al. (2017). This study uses these theoretical lenses to deepen our understanding of legal versus perceived ethical behaviour in large organisations.

The first two sections provide the practical and theoretical background against which our case study is considered, dealing first with the COVID-19 context and then with stakeholder theory, including decision-making in organisations. Then, we explain the sources and methodology used in the study, while the following section sets out our findings. Finally, we include the discussion, limitations and scope for further work.

## Background to the Australian JobKeeper Scheme

COVID-19 was first identified in December 2019 in Wuhan, China, and was declared a pandemic in March 2020, having an extraordinary impact on global economies and health (Douglas et al., 2020). The economic impact was sufficient to trigger a worldwide recession (Zumbrun, 2020). Much discussion has occurred about the best way to help businesses recover from and overcome the COVID-19 crisis (Donthu & Gustafsson, 2020; Fakhruddin et al., 2020; Manuel & Herron, 2020). Many countries, including the United States, Canada, New Zealand and Australia, implemented schemes to support business and the economy (OECD, 2020).

The COVID-19 pandemic has been seen by many as a crisis (Cassells & Duncan, 2020). Crises are unexpected, impactful, and disrupt business activities (Bundy et al., 2017) and businesses commonly perceive crises as adversities (Williams et al., 2017). Ethical considerations are heightened in times of crisis where there is the “perception that an individual or set of individuals faces a potentially negative outcome unless some type of corrective action is taken” (Dutton, 1986). The challenging circumstances may put additional pressure on companies and their ethical decision-making.

The JobKeeper scheme was a payment given to Australian companies affected by COVID-19 as a means to support employment. When JobKeeper began in March 2020, the payment was A$1500 per fortnight per employee (or about US$1200), which was roughly on par with average weekly earnings. To be eligible, a recipient enterprise had to show that its predicted revenue for a defined period (either 1 or 3 months) would be at least 30% lower than for the same period a year earlier, 50% lower for firms with turnover in excess of A$1 billion.

A ‘large company’ is an actively trading business with more than 200 employees (ABS, 2020). Large companies are included in the official list of a prescribed financial market (for example, listed on a securities exchange such as the ASX), and shares can be bought/sold by members of the public. We limit the study to large companies for two reasons. Firstly, large companies publish financial statements and other financial information, such as CEO bonus payments, which journalists and business analysts can review.

Furthermore, large organisations would be less subject to external disruption as it is easier for larger organisations to allocate resources to plan and handle crises (Parnell & Crandall, 2021), and they have better access to information (Herbane, 2013; Kurschus et al., 2017) and are participating in the accelerated digitalisation process (Amankwah-Amoah et al., 2021). Therefore, observing large organisations’ behaviours under crisis pressure offers a special opportunity to explore perceived ethical and unethical behaviours. In setting this limit, we are consistent with the extensive review of stakeholder reactions carried out by Henisz and his colleagues, who say that the risk of missing pertinent actions or statements is “further mitigated” by restricting the analysis to large companies whose actions are “strong candidates for inclusion in the industry and national media” (Dorobantu et al., 2017, p. 571).

After 6 months, all payments were reviewed, and a new test was applied based on actual revenues; the scheme ended in March 2021 (Australian National Audit Office, 2022). Some large Australian companies with annual revenue in excess of A$1 billion dollars received payments, and there was much public comment when some of these companies later reported their results and disclosed increased profits and payments to directors and senior executives (Crowe, 2021). Much of the public comment had ethical content, as we show below. The Australian Tax Office (ATO) assessed all applications, and there is no suggestion that any company acted illegally, although some suggested that there was an ethical obligation to behave differently (Ore, 2021). Although differences in reporting periods complicated forecast and actual revenues comparisons, this is of little significance for our public perception and pressure study. The perception was that JobKeeper was to help those who would be adversely affected by the crisis, and an increase in profit was seen to indicate that there had been no adverse effect.

## Stakeholder Theory

Stakeholder theory (Freeman, 1984) provides a useful theoretical framework for considering the government’s stimulus actions to overcome employment problems caused by the COVID-19 crisis. Stakeholders are “any individual or group who can affect or is affected by the actions, decisions, policies, practices or goals of the organisation” (Carroll, 1991, p. 1). It has previously been used as a framework in ethics research (Harris & Freeman, 2008; Laplume et al., 2008). The traditional description of stakeholder theory, as expressed in the widely-cited Mitchell et al. (1997) paper, includes a section on the identification of stakeholders, showing that firms should pay attention to stakeholders who exhibit power, legitimacy and urgency. A stakeholder that has all three attributes can be considered a stakeholder of high salience, one to which “managers give priority” (Mitchell et al., 1997, p. 878). Stakeholder theory is relevant to our consideration of how large organisations and the community have responded to the JobKeeper programs, and it is particularly relevant to investigating how a firm responds to external pressure. Thus, we consider how organisations responded to the COVID-19 crisis policies and how they consequently responded to public pressure to repay government support grants when the public viewed the fund provision as unnecessary. We focus on Australia and the response of large companies that received JobKeeper grants from the Australian Government.

A key plank of stakeholder theory is that companies should pay attention to stakeholders. Much effort has been devoted to determining and describing the ways in which firms respond to stakeholder pressure. Businesses respond if they perceive a need to protect their interests (Rowley & Moldoveanu, 2003; Winn, 2001; Wolfe & Putler, 2002) or trigger a reaction from the business perspective (Jones, 1995). Businesses can only gain stakeholder support by creating a trusting and not opportunistic environment (Heugens et al., 2002; Hosmer & Kiewitz, 2005). Sometimes businesses respond to public pressure with charitable contributions (Brammer & Millington, 2004), reputation management, and so on (Ulmer & Sellnow, 2000). A crisis often triggers a business response (Dorobantu et al., 2017).

Most descriptions of the stakeholder theory include multiple stakeholders, and the multiplicity of stakeholders and their interrelations have been the basis of comments about the complexity of the theory considering the stakeholders’ actions and reactions (Dorobantu et al., 2017). To allow us to focus on the way in which stakeholder theory provides a useful framework for analysis of the behaviour of firms with regard to JobKeeper payments, we have chosen to focus on the firm (the entity at the core of most traditional stakeholder diagrams) and a group we will call the general public. The stakeholder list remains extensive; the simplification does not reduce the inter-relationships but allows the factor of time to be considered as the salience of the general public changes as information becomes available and as the behaviour of some stakeholders affects the behaviour of others.

This time-sensitive effect is shown through public opinion. The general public can influence business leaders through public opinion (Dorobantu et al., 2017; Nartey et al., 2022), which is defined as “an aggregate of individual views, attitudes, and beliefs about a particular topic, expressed by a significant proportion of a community” (Davison, 2022). The importance of public opinion in influencing business behaviours has been recognised previously and is often called “the court of public opinion” (Ruggie, 2008). The stakeholders receive information and decide to take public action based on that (Nartey et al., 2022). Ethical standards can move quickly (Ferdowsian & Beck, 2011), as can perceptions and attitudes (Drinkwater et al., 2019). As stakeholders, the general public can influence the actions of companies by expressing their perception of the situation and promoting a debate about the ethical decisions of companies. Organisational values that are opposite to public opinion are risky and can create conflicts. This situation may also introduce additional costs for businesses and affect their reputations and financial performance (King & Soule, 2007) by influencing the company market value (Nartey et al., 2022). Social media also makes the transmission and spread distribution of the public perceptions much faster than before.

Organisations regularly undertake decision-making as a critical business process (Mumford et al., 2007), which may involve ethical components (Treviño et al., 2006; Zeni et al., 2016). Perhaps one of the most widely referenced models of ethical decision-making is the four-component model of Rest (Rest, 1984), which has been adapted many times (Haidt, 2001; Werhane, 2008). For this study, the core components—awareness, judgement, intention, and behaviour—are applied as in the issue-contingent version proposed by Jones (1991).

## Practical Focus of the Study

This research focuses on the practical perception of ethical violation as opposed to a normative analysis of business ethics during the pandemic. Whilst the health and economic aspects of the crisis were quickly recognised, the behaviour of corporations, particularly in the way they handled government support packages, suggested that there might be an ethical element to the crisis, distinct from the ethical issues associated with vaccine distribution and trade-offs between individual freedoms and community health benefits. The COVID-19 pandemic is a crisis, and ethical behaviour is threatened in a crisis. Jones (1991) suggests that the nature of an ethical issue will affect the ethical decision-making process. This can be applied to the COVID-19 crisis. The Australian community were forced to confront a debate about the perception of ethical behaviour—when something legally permitted might still be perceived as ethically wrong by stakeholders. This study explores how far the community, especially stakeholders, recognised this distinction between legal and ethical action.

## Methodology

This is exploratory research because while we have issues to test/direct and focus on our task, we do not seek to test quantitative relationships. Our primary purpose is to examine the response of large organisations to the JobKeeper support program, to show how this triggered diverse behaviours under the influence of stakeholders, and to show that the COVID-19 crisis was an ethical as well as a health and economic crisis.

We embarked on a case study. Media coverage of the various ways large organisations handled the JobKeeper payment alerted us to some of the ethical aspects of both the behaviour and the commentary. The initial, informal review of the media reports suggested that complex organisational issues were involved. The complexity was reinforced by our understanding that ethical decision-making is an interdisciplinary field that has been the subject of study by philosophers, psychologists, sociologists and neuroscientists, among others.

The case study approach is appropriate where complex organisational phenomena are involved and “allows an investigation to retain the holistic and meaningful characteristics of real-life events” (Yin, 1994). We were certainly dealing with real-life events, and the variety of responses apparent from the initial inspection of the media reports suggested that complex organisational issues were involved. The implication of Jones’s (1991) issue-contingent model, that there was no clear boundary between phenomenon and context, between ethical behaviour and the issue that prompted it, meant that another of Yin’s (1994) criteria for using a case study was met.

We chose to use secondary data rather than collect data through interviews or questionnaires or conduct experiments or simulations. In the case of empirical business ethics research, the use of secondary data has several advantages, including reducing response bias by avoiding the unwillingness to respond to ethics-related questions, reducing the impact of any problems of recall, and lowering the cost of collection (Hakim, 1982, 2000). The practical example of JobKeeper and the repayment of subsidies is something that happened at a particular time in the past. Interviews may not provide accurate data about how people felt or reacted at an earlier time. Reports in the press were a valid source of information. Asking agents how they would respond to a situation described in a scenario, even when carefully prepared, will often omit elements that the agent considers important, thereby removing some of the complexity of the real world (Savur et al., 2018). The strong methodological reasons for using secondary data in business ethics research have been accepted for decades (Cowton, 1998; Harris, 2001; Nicholson & Bennett, 2009), and secondary data sources are widely used in this type of research (Saunders et al., 2007).

We used content analysis to deal with secondary data, specifically newspaper articles on the behaviour of large companies concerning the JobKeeper program. Harris (2001) stated that content analysis is a good technique for analysing beliefs, organisations, attitudes, and human relations. The analysis of the newspaper articles included coding and categorising using an Excel spreadsheet to track the coding decisions and the rationale behind them, to facilitate inter-researcher reliability and validity. The process involved coding ethical and legal aspects of the JobKeeper events from news media articles. First, we identified all the words (by definition, including synonyms) and sentences that referred to the legal or ethical actions of companies which could be judged by the public. The approach allowed us to identify the perceived legal and ethical aspects of the large corporations’ behaviours around the JobKeeper scheme over a defined period. A pilot test was conducted with 20 articles to find agreement before continuing with the complete analysis. The researchers assessed validity, revised the categories, and created a coding scheme. Finally, the calculation of the reliability indexes was applied. A generally similar method in analysing corporate responses to stakeholder action was used by Henisz and his colleagues (Henisz et al., 2014).

### Australia as a Case Study

Three features make Australia an appropriate location for a case study of ethical behaviour by large organisations during the COVID-19 pandemic. The first is its relative isolation—an island nation able to close its borders effectively. The second is the size and developed nature of Australia’s economy—Australia is a member of the OECD. Australia also has well-established governance institutions, a well-respected media landscape, including newspapers, and high literacy levels. The JobKeeper program itself is the third reason why Australia provides a valuable case study to examine the ethical aspects of corporate behaviour during a crisis. JobKeeper was a (federal) Australia-wide program that was different to pandemic assistance programs implemented in other jurisdictions (for example, individual states), and the form in which it was implemented meant that as time went by, ethical issues were raised by both the recipients and the community. To address the explored issues, we examine the community response disclosed in the mainstream media. As it reported on the progress of the COVID-19 pandemic and the responses of governments and companies, the major outlets of the Australian press also carried comments on corporate behaviour.

Wanting to conduct a methodologically rigorous study that would produce “evidence-informed management knowledge”, we adapted Tranfield et al.’s (2003) three-stage approach to systematic literature reviews: planning the review, conducting the review, reporting and dissemination. A summary of the process we used is provided in Table 1. Validity, reliability and reproducibility (Higgins et al., 2019) are enhanced by the explicit description of the process used to guide the sample selection and the qualitative analysis. That Iden et al. (2017) and Bhimani et al. (2018) also used this process heightened our confidence in the validity and reliability of the methods we had adopted.

**Table 1:** Overall review process, after Tranfield et al. (2003)

Stages of the review*	Process	Outcome
Planning • Identification for the need for a review • Preparation of a proposal for a review • Development of a review protocol	Develop clear research questions arising from the literature Select a source of information	JobKeeper as a case Explore: the perceptions of ethical wrongdoing in large organisations (from the public opinion perspective), which does not mean that companies break the law Australian newspapers as an information source
Conducting • Data extraction and monitoring progress • Data synthesis	Content analysis Show the link between evidence (from content analysis) and ethical behaviour (from literature & models)	Content analysis Focus on JobKeeper and large organisations
Reporting and dissemination • The report and recommendations • Getting evidence into practice	Publication in relevant academic journals and presentation at management and professional conferences	This paper

*Sub-stages are based on the phases listed in Fig. 2 in Tranfield et al. (2003)

The COVID-19 pandemic was a significant news story around the world and Australia was no exception (Jack, 2021). Australian newspapers are a source of information on the response of large organisations to the JobKeeper payment. They also provide information about community reactions. Content analysis was guided by a protocol based on the process outlined by Krippendorff (1980). These steps include selecting the texts to be analysed, choosing the analysis unit, and constructing a ‘dictionary’ of search terms. The sample was restricted to newspapers published in Australia, in English, and newspapers with national circulation or a reputation for business and financial affairs coverage. Only those with full text available were included in the sample. An effort was made to provide balanced coverage by including items from the two major media groups operating in Australia and both sides of the political divide. We used two comprehensive news article databases: ProQuest (Australia and New Zealand news streams) and Gale OneFile News. Only articles related to JobKeeper and large organisations were included in the first selection round. The search terms used were of job-keeper and large organisations, including different spellings and synonyms. A combination of those terms appeared in multiple articles.

Our aim is to examine the interaction between firms and their stakeholders, particularly the reaction of large organisations to the general public as a stakeholder. As we aim to discover or describe what it is that the community thinks as a stakeholder, the search excluded items from newspapers that could be classified as opinion, correspondence, letter to the editor, interview, or editorial, as these could be considered personal views, or items seeking to influence public opinion and thus be less representative of public opinion. Indeed, many opinion pieces and editorials are written with the intention of changing public opinion rather than representing it.

**Fig. 1:**
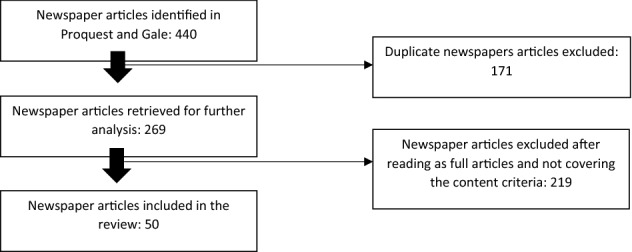
The newspaper sample

The search was undertaken on 24 November 2021. In the first round, we retrieved 266 articles from Proquest and 173 from Gale—a total of 440 newspaper articles. We removed newspaper articles that were duplicated within the same databases (e.g., print and online versions or multiple outlets with shared services) and those that were duplicated across both databases; 171 were deleted from the analysis at this stage and 219 in the next stage (see Fig. 1), resulting in 50 newspaper articles for more detailed content analysis. The sample was reduced by another 219 by removing news items that dealt with nonrelevant matters, leaving 50 newspaper articles for more detailed content analysis. This process is shown diagrammatically in Fig. 1.

### Analysis of the Items

To apply content analysis to our study, we selected a list of terms that could be used to describe a range of ethical norms. The phrase was selected as the unit of analysis. Initial assumptions for the coding process were developed (Thornberg & Charmaz, 2014), and a list of terms that could reasonably be said to encompass the field of business ethics was developed using previous knowledge of business ethical concerns, using ethics definitions, synonyms and antonyms. The researchers discussed the coding scheme, the emerging data from the content analysis, and the categories based on the theoretical construct presented above during the course of the data collection to find agreement before continuing with the complete analysis. A list of the ethics, synonyms and antonyms is presented in Table 2, including the word count of those selected terms. The final selection of words and phrases deemed to infer ethical behaviour included transparency, privacy, inequity and unfairness, the legal environment, Corporate Social Responsibility (CSR), lying and honesty, power, and conflict of interest.

**Table 2:** Summary of ethical concerns findings

Business ethics definition	“Business ethics refers to the standards for morally right and wrong conduct in business”					
Related words	Synonyms/antonyms	Word count frequency	Topic	Definitions*	Articles**	Exemplary quotes
Compliance	Code—legislation—rule—regulation—principle	Right (205)—Rules (86)	Compliance	“A company’s or a business person’s conformity with relevant laws and regulations”. MacDonald, C and Marcoux, A (2022)	1, 31, 32, 37, 38, 39, 42, 62, 90, 100, 115, 121, 147, 149, 150, 151, 167, 170, 184, 210, 222	“There is no legal requirement for companies to hand back funds they were paid under JobKeeper. Treasurer Josh Frydenberg has defended the scheme, saying requirements to return funds would have delayed emergency payments and damaged the economy”. (39)
Honesty—integrity—honor—decent	Goodness—morality—goodwill—virtue—value—true—good- ethical	Good (115) integrity (24)—True (16)—Bad (20)—Scandal (16)—Wrong (8)—Goodwill (4)	Lying and honesty	Honesty is: “always telling the truth, and never stealing or cheating” (Oxford English Dictionary, 1989)	28, 121, 149, 254, 263	“The Tax Office is sifting through thousands of tip-offs about businesses enrolled in the JobKeeper scheme allegedly ripping off employees”. (254)
Lie	Dishonest—false—wrongdoing			Lying is: “to make a false statement with the intention to deceive” (Oxford English Dictionary, 1989) “A lie is a statement made by one who does not believe it with the intention that someone else shall be led to believe it”,(Isenberg, 1965) “Lying is making a statement believed to be false, with the intention of getting another to accept it as true”.(Primoratz, 1984)		
Power	Influence—capability	Conflict of interest (95)—Power (65)	Power	“the ability to control people or things” (Oxford English Dictionary, 1989)	25, 28, 39, 59, 78, 93, 100, 164, 211, 218, 254, 263	“If your firm is getting taxpayer assistance, the boss shouldn’t be getting a bonus, and shareholders shouldn’t be getting stonking dividend”. (100)
			Conflict of interest, CEO bonuses and dividends	“A person has a conflict of interest if a) he is in a relationship with another requiring him to exercise judgement in other’s service and b) he has an interest tending to interfere with the proper exercise of judgement in that relationship” (Davis, 1993, p. 21)	1, 25, 28, 34, 39, 40, 63, 74, 77, 78, 81, 90, 93, 121, 149,150, 162, 164, 191, 210, 218, 222, 229	“New analysis by the Parliamentary Budget Office has found that at least 42 per cent of the government’s $89bn JobKeeper program was directed to businesses where turnover did not fall below threshold limits during the quarter, they claimed financial support”. (150)
Transparency	Transparency—safe—clear—trust	Respect (54)—trust (44)—Transparency (10)—Safe (4)	Transparency	“Transparency is a widely shared but vague term broadly referring to information disclosure as a key to better governance”.(Valentinov, Verschraegen, & Van Assche, 2019, p. 289) In business ethics and information ethics, ‘‘transparency tends to be used to refer to forms of information visibility, which is increased by reducing or eliminating obstacles… possibility of accessing information, intentions or behaviours that have been intentionally revealed through a process of disclosure”. (Turilli & Floridi, 2009, p. 105)	1, 42, 148, 164, 177, 199, 210, 222	“JobKeeper was extraordinarily generous but accompanied by very little transparency, and the fact of the matter is that the Australian taxpayer will be paying for employers who’ve been unjustly enriched by JobKeeper for the next 30 years,” Mr Paatsch said. (210)
Equitable & fair	Fair—decent—(antonisms inequity)	Fair (39)—Inequity (4)	Inequity and fairness	The Federal Trade Commission (1994) amended definition “The act or practice causes or is likely to cause substantial injury to consumers; which is not reasonably avoidable by consumers; and is not outweighed by countervailing benefits to consumers or to the competition”. (Beltramini, 2003, p.394)	1, 28, 93,100, 164, 197, 222	“It ain’t fair. Jobkeeper was meant to save the jobs of battlers, not line the pockets of billionaires”. (100)
Privacy	Privacy—secrecy—Disclosure—breach	Secrecy (22)—Privacy (20)—Breach (20)—Disclosure (3)	Privacy	A common theme is that privacy refers to information control (Altman, 1976). Westin (1968), for example, suggests that privacy is “the claim of individuals … to determine for themselves when, how, and to what extent information about them is communicated to others” (p.7). Also, privacy can be understood as the individual’s and organisation’s rights about what, when, and how information from them is shared (Udo, 2001)	1, 14, 56, 85, 162, 163, 177, 199, 210, 218	“But while the US and New Zealand governments maintain public databases of which companies receive their wage subsidies—and how much—the Australian government has chosen to keep jobkeeper payments secret”. (162)
Corporate Social Responsibility (CSR)	CSR—corporate social responsability	CSR (4)	CSR	“businesses seeking to improve society, the community, or particular stakeholder groups” (Carroll, 2016, p. 1)	85, 133, 210, 222	“Perceptions of humanity—demonstrating genuine care for those affected by an organisation’s operations—has been the weak spot for most companies in recent years.” (133)

*Source: Altman (1976). Privacy: A concept analysis. *Environment and Behaviour, 8*(1), 7–29. Beltramini, R. F. (2003). Application of the unfairness doctrine to marketing communications on the internet. *Journal of Business Ethics, 42*(4), 393–400. Carroll, A. B. (1991). The pyramid of corporate social responsibility: Toward the moral management of sorganisational stakeholders. *Business Horizons, 34*(4), 39–48. Davis, M. (1993). Conflict of interest revisited. *Business & Professional Ethics Journal*, 21–41. MacDonald, C., & Marcoux, A. (2022). Corporate Citizenship. *Concise Encyclopedia of Business Ethics*. ISBN 978-0-9940760-1-4. Retrieved from March 10, 2022, from http:www.conciseencyclopedia.org. Oxford English Dictionary. (Ed.) (1989). Oxford: Clarendon Press. Turilli, M., & Floridi, L. (2009). The ethics of information transparency. *Ethics and Information Technology, 11*(2), 105–112. Udo, G. J. (2001). Privacy and security concerns as major barriers for e‐commerce: a survey study. Information management & computer security. Valentinov, V., Verschraegen, G., & Van Assche, K. (2019). The limits of transparency: A systems theory view. *Systems Research and Behavioral Science, 36*(3), 289–300

**Words can be repeated multiple times in an article

The NVivo 12 application was used to analyse and code the data. Steps were taken to assess validity, reliability, and reproducibility at various points of the process. A test coder was provided with a sample of 10 excluded items, 10 included items and the coding scheme and asked to judge which should be included in the analysis; there was 77% agreement between the test coders (3) and the study sample. The test coder conducted multiple meetings regarding the items identified as ethical issues until a satisfactory agreement was reached. These results testify to the validity, reliability, and reproducibility of the content analysis undertaken in the study.

## Findings

Table 2 shows the number of times each concern was recorded, grouped together under the headings compliance; honesty, integrity, honour, decent; lie; power; transparency; inequity and unfairness; privacy; and CSR, along with the article in which it was observed, and an example of the wording which supported the finding. Providing these examples, which come from eight distinct articles in eight newspapers, is an element in the validation of the content analysis exercise, helping to establish the face validity of the analysis.

### The JobKeeper Program and Ethical Concerns

Table 2 shows that, based on our sample of newspaper items mentioning Jobkeeper, items clearly related to ethics appeared 876 times in total. Thus, COVID-19 was clearly seen as an ethical issue. Some might argue that we have shown only that there was a perception of an ethical issue. That may be so. To do otherwise would require a rigorous test of ethical behaviour and we argue that there are both legal and communal elements in the definition of ethical behaviour. Neither we, nor any other commentator we have found, nor any member of the general public has argued that the failure to repay JobKeeper involved legal wrongdoing (please refer to Appendix 1 for the final selection of news articles and ID).

### The Role of Public Pressure on Firm Behaviour

Our second aim was to examine the role of the general public as a stakeholder and the impact the public has on a firm’s behaviour. In this regard, we rely first on the data presented in Table 2 and second on a more detailed analysis of the behaviour and public comment related to an individual firm.

The practical question is one of salience or of how a firm determines which stakeholder(s) it must give attention to. Mitchell et al. (1997) proposed that three factors—power, legitimacy, and urgency—influence the attention that a corporation gives to a stakeholder. We contend that the finding demonstrates the legitimacy and power of the general public as a stakeholder and that the public perceives an ethical element in the behaviour of some businesses with regard to JobKeeper. Not only is their behaviour subject to ethical examination, but also the general public is the appropriate body to do the examination. That gives the general public power, legitimacy and urgency. Further use can be made of the data underpinning Table 2 to show how actions by the general public as stakeholders “provided information signals” to executives, investors and analysts (Dorobantu et al., 2017, p. 563).

In the case of JobKeeper, public pressure triggered multiple reactions in organisations. This was especially true for companies that later reported improved performance: some returned the money, some kept it as they were legally entitled to do, and some returned part of the money. Notably, media comment extended across the spectrum of corporate responses—return, keep, and partial—and was coupled with ethical comments. Evidence of both the pressure and the various reactions were found in our study.

The situation unfolded over about 12 months from the inception of JobKeeper. After the media reported that an independent analysis had found that $13 billion was paid to companies with increased revenues, the JobKeeper program came under further scrutiny, and public pressure rose (149). Some requested that the public receive information about which companies had profited (150, 164, 56) and how much money was paid in executive bonuses (150, 164) and dividends (222) during this period. Furthermore, the media pointed out double standards of companies talking about CSR but using money they do not need, and which could help others instead (210). Those companies abused the goodwill of the taxpayer (218), and the Council of Small Business Organisations Australia called out the big firms for their disgraceful behaviour during the pandemic, saying that those organisations do not have much regard for Australia (229). Furthermore, the Business Council of Australia (28), a lobby group for Australia’s largest companies, blasted unacceptable behaviours around the payment of bonuses and dividends (28) and asked the businesses to pay them back (229). Furthermore, the ATO noted that some organisations have been using artificial mechanisms to exploit the system (25), with a commissioner pointing out that firms should “follow the tax law, but also follow the spirit of the law” (25) and that using funds from a scheme with a different purpose “to support executive bonuses, increase dividends, or repatriate cash to offshore related parties is likely to be viewed poorly by the community” (25).

The ability of large businesses to maintain community trust and deliver integrity and humanity would be called into question by such behaviour, according to a KPMG working paper mentioned in the media (133). The need for clearer reporting of JobKeeper payments was raised by many, including investors and regulators (81). Some comments recognised the complexity, with an investor recognising that JobKeeper kept employees in their jobs but also benefited shareholders that were already quite well off (81) but avoided answering the question about whether it is wrong or opportunistic behaviour (81). The government was also criticised, with comments like “the wildly expensive JobKeeper will rank as the single most irresponsible and reckless spending program ever undertaken by a government” (1) and “the biggest waste of public money in living memory” (63). Both positive and negative views are consistent with the findings of the Henisz studies regarding “stakeholder-initiated action” (Dorobantu et al., 2017; Henisz et al., 2014). Multiple questions arose when the JobKeeper program was extended, and there was a lack of support from the community of economists (155); some would have liked to have seen additional criteria for access to the JobKeeper program (such as income tests for partners) (155). Furthermore, there was also some push for the Government to recognise that not all the funding was correctly used, and some organisations were paying bonuses and dividends (164).

This pressure and public concern about corporate behaviour came at a time when the JobKeeper support program was seen as successful on balance. The Prime Minister (164) supported the results of JobKeeper, as he believed that it achieved the objectives (164), and for the Federal Treasurer, JobKeeper was shown to be fit for purpose by saving jobs and aiding recovery (77). The OECD (2020) noted that job retention schemes such as JobKeeper “helped contain the employment and social fallout of the COVID-19 crisis and avoid massive layoffs”. University of Melbourne professor Jeff Borland stated that it was “the right policy for the time and in the circumstances” (90). The Australian Chamber of Commerce pointed out that JobKeeper was a “game-changer” (197), while others minimised the effect on public finances (210).

Some organisations made profits and decided to return the funding, including Super Retail Group, Toyota, Iluka, Nine and Coca-Cola Amatil, saying that the recovery was better than expected (78, 210, 229). However, some companies’ boards stopped the goodwill from these organisations (210), and some firms tried to argue that they would keep the money in case of another economic crisis (78). Harvey Norman was one of the organisations that returned part of the money after reporting surging revenue profits. It repaid $6 million in JobKeeper subsidies (149), although it initially resisted doing so (148, 210), only capitulating under public pressure to be transparent (39). Harvey Norman was paid a further $14.5 million that went to privately owned franchises, which was not returned (149). The media also pointed out the greediness of the proprietor, Gerry Harvey (148). We examine the Harvey Norman case further below. Other organisations who repaid part of the money included Premier (39, 148) and Cochlear (hearing implant provider). The latter returned $23.1 million but retained $10.4 million (39).

Many organisations received millions of dollars of public funding and refused to return the money (39), for example, Tabcorp, which received a total of $12 million (39), Eagers Automotive (received $130 million) (39), Accent (received around $24 million) (39), and BestandLess ($42 million in JobKeeper payments).

According to the business ethics literature, a firm may suffer the same consequences of negative press or public opinion in terms of perceived wrongdoing, regardless of whether the firm actually did something legally wrong or not (King & Soule, 2007). Through this study, we identified three types of responses from businesses: those who returned the funding, those who decided to keep the Jobkeeper funding and those who partially returned the money. Although all these options were legally permissible, in some cases even ethically justifiable under some ethical frameworks, public opinion played an important role. Negative public opinion arguably became more costly for these firms if they decided to keep the money than if they had given it back. For example, Harvey Norman used the middle approach, partially returning funding after the public became aware of the issue, as it was the company most frequently mentioned in the media. Yet the material published in the media shows that, despite the perception of an ethical issue, Harvey Norman’s initial judgement was to repay nothing. Public pressure continued, and Harvey Norman, “under sustained public pressure” (149), repaid some of the subsidies it had received. Whether this shows that Harvey Norman engaged in ethical behaviour is open to question. There is far less doubt that public pressure, and the intensity of the ethical issue, influenced Harvey Norman’s decision-making and its response to stakeholders.

## Discussion

The pandemic has been an important part of world life for more than two years. It has been recognised as a health and economic crisis. Businesses have faced incredible disruptions to their activities during this period, and many have been forced to close. However, as in any crisis, opportunities have also arisen, and many businesses have taken advantage of the situation. Governments worldwide have implemented support programs to reduce the impact of the crisis and consider the situation’s economic and health aspects. However, when the support programs were created, the ethical aspects and consequences linked to them were seldom considered. Our results indicate that multiple ethical concerns about business behaviours were identified after the JobKeeper support program started. Our study was limited to large organisations because while all organisations have faced constraints during the pandemic, large organisations have had some advantages over SMEs. We have been able to demonstrate that ethical concerns arose from the behaviour of large organisations during the COVID-19 pandemic. Thus, the crisis is an ethical crisis as well as a health and economic crisis.

Our research specifically analysed the ethical aspects without considering the health and economic aspects at this point; although they are highly related, the economic aspects directly influence ethical decisions. Our research was a deep dive into the ethical aspects and found that they can be subdivided into two streams—context and organisational related.

Previous knowledge was used as a baseline to identify ethical concerns. We analysed the concerns from the context and organisations’ attitudes and behaviours in more detail. Ethical issues arose as a consequence of four contextual aspects: transparency, privacy, inequity and unfairness, and compliance. We found that conflict of interest affecting ethical decisions was the aspect most considered in the press. The second part of our analysis showed the impact of public pressure in identifying unethical behaviours and how businesses readjusted their behaviours. COVID-19 has been regularly described as a health and economic crisis. We have shown that the public perceived a significant moral content and that Harvey Norman, for one, and probably all the firms who openly said, “It was legal, and we will not give any back”, or only returned partially funding after public pressure, it is a perception of acting unethically.

This research opens a debate about meeting stakeholders’ expectations and the consequences businesses may face if they do something that conflicts with these expectations. Although it is the first step, a legal justification may not be enough from the managerial perspective. Considering the ethics of a decision and how stakeholders perceive it would help businesses to face further conflict. It is important for organisations to consider the impact of their ethical behaviours and how they it will impact their performance. The public pressure did affect not only the context (as the Government changed the rules for the second round of JobKeeper funding) but also the large organisations’ ethical behaviours once they were exposed and public pressure was established.

From the business perspective, ethical behaviours, especially in times of crisis, are scrutinised by society. A link between business and society is included in the theoretical bases of both business and ethics (Elkington, 1999). The lack of recognition of the importance of being on the legal and ethical side may affect organisations’ relationships with the community. Our study has shown that conflict of interest was an important aspect, possibly recognising the temptations of managerial leaders to make wrong decisions when personal interests are involved.

## Limitations and Future Research

The COVID-19 pandemic was unprecedented, the JobKeeper program has not been tested thus far, and it is hard to find a baseline program for comparisons. Other newspapers could also be added to complement the data; however, the main sources showed that repetition was a common factor in other outlets. This might limit extension to other countries where different support packages may have been introduced.

We should also acknowledge that we relied on secondary data, not direct interviews with the actors involved, and this is a source of limitation in the data source. Future research may include interviews with direct actors from large organisations who received the JobKeeper subsidy and acted in a perceived unethical manner in the face of public scrutiny. In addition, this paper focused only on ethical and legal standards, leaving other standards out of the debate, such as religious, professional, and industry, among others that may affect decision-making. This opens the opportunity for further research with the introduction of other standards. Furthermore, as there are differences between large organisations and SMEs regarding resources and ways of responding to crises, a comparative analysis of their ethical behaviours may increase knowledge of those differences. Further research on business behaviours could be done by investigating the responses of SMEs once their perceived unethical business behaviours come under public scrutiny (three types of responses were identified but not deeply investigated). Further research could investigate those organisations’ responses in more detail. Although, as our sampling began with a broad search, there may be little more to find.

## Appendix 1: Data Included in the Analysis

**Table:**

ID	Database	Title	Date	Publication
1	Proquest	$13bn went to firms with rising revenue	Sep 22, 2021	The Australian (Online)
14	Proquest	ALP’s JobKeeper push opens a second front to breach tax secrecy	Oct 24, 2021	The Australian (Online)
25	Gale	ATO warns businesses not to use loopholes to exploit $30bn Covid tax concessions	Oct 29, 2020	The Guardian
28	Gale	Australian companies getting jobkeeper shouldn’t be paying bonuses	Sept 6. 2020	The Guardian
31	Proquest	‘Awful decision’: Business urges JobKeeper overhaul: Exclusive	Apr 20, 2020	Sydney Morning Herald
32	Proquest	Ballet makes $15 m bid for survival: The Arts	May 16, 2020	The Age
34	Proquest	Bendigo JobKeeper: Businesses call for extension as economic cliff looms	Mar 11, 2021	Herald Sun (Online)
37	Proquest	Big business will be hunting for bargains in the pandemic wreckage	Dec 4, 2020	The Australian (Online)
38	Gale	Big business workers heading for dole	Aug 4, 2020	The Australian
39	Proquest	Big companies keep payments despite profits	Sep 4, 2021	Sydney Morning Herald
40	Gale	Big companies reap JobKeeper rewards	Aug 12, 2020	The Australian
42	Proquest	Bleeding behind the stage curtain	May 16, 2020	The Age
43	Proquest	Blockbuster movies help cut Event’s loss	Aug 24, 2021	Herald Sun
56	Proquest	Business beneficiaries should be named: poll: Exclusive	Sep 24, 2021	Sydney Morning Herald
59	Proquest	Business urged to step up for ‘Team Australia’	Apr 29, 2021	The Australian
62	Proquest	Business vows to save jobs after massive subsidy	Mar 31, 2020	Sydney Morning Herald
63	Gale	Businesses that had no downturn from Covid crisis received $12.5bn jobkeeper windfall	July 23, 2021	The Guardian
74	Gale	Christian Lobby got JobKeeper despite surges in revenue	Sept 6. 2021	The Age
77	Proquest	Clawing back JobKeeper ‘a risk to recovery’	Oct 12, 2021	The Australian
78	Proquest	‘Close to theft’: small business slams wage subsidy profits: Exclusive	Mar 6, 2021	Sydney Morning Herald
81	Gale	Companies using millions in jobkeeper payments to pay increased dividends to shareholders	Aug 11, 2020	The Guardian
85	Proquest	Confusion over Jobkeeper	Sep 2, 2021	The Australian
90	Proquest	Coronavirus: JobKeeper clawback ‘would risk recovery’, says Treasury	Oct 11, 2021	The Australian (Online)
93	Proquest	Crossbench senator shuns Coalition over payments	Sep 6, 2021	The Age
100	Proquest	Fairness is another casualty	Sep 15, 2020	Herald Sun
115	Gale	Generous JobKeeper gave us a very good virus	Mar 26, 2021	The Australian
121	Proquest	Greens to call in JobKeeper ‘debts’	May 3, 2021	Herald Sun
133	Proquest	How companies can maintain trust	Aug 3, 2020	The Australian
147	Proquest	JobKeeper buck stops with Treasury boss	May 30, 2020	Sydney Morning Herald
148	Proquest	JobKeeper dents licence to give health advice: Comment	Sep 9, 2021	Sydney Morning Herald/The Age
149	Proquest	JobKeeper for Christian group with cash rise	Sep 6, 2021	Sydney Morning Herald
150	Proquest	JobKeeper forked out $38bn to thriving firms	Nov 2, 2021	The Australian (Online)
151	Proquest	Jobless now collateral damage in culture war	May 15, 2020	The Age
155	Proquest	Jolting the system	Oct 4, 2020	Sunday Age
162	Gale	Labor demands 200 big companies reveal if they used jobkeeper to pay dividends	Oct 18, 2020	The Guardian
163	Proquest	Labor must come to its senses over tax secrecy	Oct 26, 2021	The Australian
164	Proquest	Labor pursues businesses cashing in on JobKeeper	Feb 15, 2021	Herald Sun
167	Proquest	Limit big redundancies or nation will face huge bill: business group	Feb 8, 2021	Sydney Morning Herald
170	Proquest	Matt Canavan urges firms to help as ‘NationKeeper’	Apr 28, 2021	The Australian (Online)
177	Proquest	Most want JobKeeper companies revealed	Sep 24, 2021	The Age
184	Proquest	No JobKeeper: big business workers heading for dole	Aug 5, 2020	The Australian
191	Proquest	One-third of businesses seeking help	Apr 11, 2020	Weekend Australian
197	Proquest	PM’s $130b lifeline for millions of workers	Mar 31, 2020	The Age
199	Proquest	PM’s trip betrays culture of creeping secrecy	Sep 10, 2021	Sydney Morning Herald and the Age
210	Proquest	Push on corporate Australia to repay JobKeeper as profits recover: Wage subsidy	Jan 26, 2021	Sydney Morning Herald and the Age
211	Proquest	Qantas wins JobKeeper appeal	Dec 17, 2020	The Australian (Online)
218	Gale	Senate backs inquiry into whether tax commissioner should release jobkeeper details	Oct 19,2021	The Guardian
222	Proquest	Senator shuns Coalition over JobKeeper payments	Sep 6, 2021	Sydney Morning Herald
229	Proquest	Small business slams JobKeeper hoarders	Mar 6, 2021	The Age
254	Proquest	Tip-offs point to JobKeeper rorts: Exclusive	Jun 14, 2020	Sun Herald
